# Editorial: Novel therapeutic strategies for chronic kidney disease: from bench to bedside

**DOI:** 10.3389/fcell.2023.1242473

**Published:** 2023-07-06

**Authors:** Chunling Huang, Xin-Ming Chen, Yongli Zhao

**Affiliations:** ^1^ Renal Research Lab, Royal North Shore Hospital, Sydney Medical School, Kolling Institute, University of Sydney, Sydney, NSW, Australia; ^2^ Second Affiliated Hospital of Dalian Medical University, Dalian, China

**Keywords:** chronic kidney disease, kidney fibrosis, therapeutic appraoches, cell therapy, cell-free therapy

Chronic kidney disease (CKD) is a life-threatening disease associated with an increased risk of end-stage renal disease, cardiovascular disease, and premature death. Due to the rise in the ageing population and its risk factors, such as obesity, diabetes mellitus and hypertension, the prevalence of CKD has been increasing rapidly in recent years. CKD is projected to become the fifth most common cause of death globally by 2040 (Foreman et al., 2018). Although current treatment regimens have shown some positive results in delaying the progression of CKD, the risk of kidney failure requiring kidney replacement therapy, as well as cardiovascular morbidity and mortality in CKD patients, remains very high, imposing enormous medical and financial burdens on patients and society. This Research Topic aims to provide an update on potential therapeutic strategies for various CKD and the new developments in a wide range of novel treatment approaches.

Kidney fibrosis is a final common pathologic feature of CKD, irrespective of the aetiology, which is characterized by excessive production and deposition of extracellular matrix components in the kidney interstitial space (Li et al., 2022). Myofibroblast is the main contributor to kidney fibrosis (Moeller et al., 2021). Although the cellular origin of kidney myofibroblasts remains an open field of investigation, resident fibroblasts are increasingly recognized as the predominant source of myofibroblasts due to the great advances in genetic lineage tracing techniques. Resident fibroblasts play a key role in the development and progression of kidney fibrosis by producing excessive extracellular matrix proteins (Moeller et al., 2021). A better understanding of the heterogeneity of kideny fibroblasts and the molecular mechanisms of fibroblast activation is favoured to discover new therapeutic approaches for kidney disease. Huang S et al. provided a contribution by investigating the role of megakaryocytic leukemia 1 (MKL1), a master regulator of myofibroblast activation (Li et al., 2019; Mao et al., 2020), in three different fibrosis models. By generating the resident fibroblast conditional MKL1 knockout mice, they confirmed that the cellular origin of myofibroblasts is predominantly from resident fibroblast transition. Moreover, they found that MKL1 deletion in resident fibroblasts alleviated kidney fibrosis, myocardial fibrosis, and pulmonary fibrosis, indicating that resident fibroblast-specific MKL1 is a key mediator to drive a pro-fibrogenic response *in vivo*.

The recent advances in sequencing technologies have notably increased our knowledge of autosomal dominant polycystic kidney disease (ADPKD), the most common genetic cause of CKD with the formation of cysts in the kidneys. Extensive genetic studies from patients and animal models have identified several causal ADPKD genes, with about 85% of ADPKD cases associated with PKD1 mutation (Lanktree et al., 2021). The comprehensive genetic testing to improve the detection rate of pathogenic genes is critical to for diagnostic and prognostic of ADPKD. Shang et al. reported a newly established system of genetic detection and analytical methods from next-generation sequencing to multiplex ligation-dependent probe amplification to targeted region sequencing and finally to Sanger sequencing. They combined multiplex polymerase chain reaction and targeted region sequencing for the first time in ADPKD diagnosis, which further improved diagnostic accuracy. They also identified one new missense mutation and four new deletion mutations using this new system.

Over the last decades, cell therapy has emerged as a novel promising strategy to modulate the progression of CKD by promoting the regeneration of damaged kidney tissue (Torrico et al., 2022). Among all cell therapy, mesenchymal stem cells-based (MSCs) therapies have attracted increasing attention due to their multifaceted therapeutic properties, including immunomodulatory, anti-fibrotic, anti-inflammation, antioxidant, anti-apoptotic, and angiogenic activity (Allinson et al., 2023; Chen et al., 2023). MSCs constitute a pool of multipotent cells with multi-lineage differentiation, self-renewal, and proliferative potential. These fibroblast-like stem cells can be derived from a diverse range of human tissues, including bone marrow, umbilical cord, adipose tissue, placenta, dental pulp, amniotic fluid, Wharton’s Jelly, lung tissue, liver tissue and dermal tissue and kidney tissue (Cao et al., 2022). Accumulating preclinical and clinical studies have demonstrated the beneficial effects of MSCs therapy in various types of kidney disease, such as lupus nephritis (Wang et al., 2022), offering an innovative treatment option for kidney diseases. More well-designed studies to identify the specific cellular mechanisms of the renoprotective properties of MSCs will provide novel insights into MSC therapy, thus enhancing the future clinical application of MSCs in kidney diseases. The study by Huang C et al. demonstrated the therapeutic effects of umbilical cord MSC (UC-MSC) in lupus nephritis. Their results showed that treatment with UC-MSC significantly improved kidney function and alleviated podocyte injury in MRL/Ipr mice, which had the similar effect with the treatment of methylprednisolone, a standard care for lupus nephritis (Mejia-Vilet and Ayoub, 2021). Additionally, they evidenced the inhibition of TGF-β1 pathway in UC-MSC-treated lupus mice, providing a novel protective mechanism of UC-MSC in lupus nephritis.

Despite many advantages and benefits of MSC-based therapy, some challenges restrict their clinical application, which includes the possibility of tumorigenesis, the risk of pathogen transmission, the difficulty in maintaining a consistent source of cells with a stable phenotype, and the possibility of antibody production (immune rejection response) after repeated administration of MSCs (Taghizadeh Momen et al., 2021; Cao et al., 2022). Extensive research is devoted to looking for alternative therapies to reduce the adverse effects of MSC-based cell therapy. Researchers have identified extracellular vesicles as a popular substitute for MSCs since the therapeutic potential of MSCs is mediated mainly by paracrine secretion of extracellular vesicles, predominantly exosomes (Cao et al., 2022). Extracellular vesicles are membrane-bound vesicles released by the majority of cell types under physiological and pathological conditions (Grange and Bussolati, 2022). Exosomes are extracellular vesicles with a size range of approximately 30–100 nm. Exosomes exert diverse biological functions similar to their origin cells via the delivery of cellular cargo such as functional proteins, metabolites and nucleic acids to recipient cells (Gurung et al., 2021). They involve a broad range of physiological processes, including immune responses, tissue repair, cell-to-cell communication, inflammation angiogenesis, and coagulation (Gurung et al., 2021). Due to their complex and unique structure, exosomes have also been used as a biomarker for disease diagnosis and prognostics (Taghizadeh Momen et al., 2021). Notable research has provided convincing evidence implicating that exosomes not only play a beneficial role in various kidney diseases but also have been proposed as the promising source of non-invasive diagnostics and prognostics biomarkers for many kidney diseases (Thongboonkerd, 2019; Taghizadeh Momen et al., 2021). Zhou et al. contributed a review on the function of exosomes and exosomal microRNA in muscles and kidneys as well as their roles in the crosstalk between kidney and skeletal muscle in CKD with skeletal muscle wasting, a common complication of CKD. The authors provided an overview of several studies evidencing the beneficial effects of skeletal-muscle-derived exosomal miRNAs in different animal models of CKD, indicating that exosomes are the key mediator in the cellular communication between kidney and skeletal muscle via transferring miRNAs in CKD. Further studies to fully illustrate the mechanisms for exosomal involvement in specific kidney diseases are required for their ultimate progression towards clinical applications.

Collectively, the research topics highlighted the recent progress in the development of new treatments for CKD. The increasing knowledge in this field will enhance the translation of research findings from the bench to the bedside and ultimately lead to the successful clinical application of new therapeutic strategies for the treatment of CKD.
